# Green-synthesized *Desertifilum* silver nanoparticles induce *BAX/BCL-2* mediated apoptosis in Ehrlich carcinoma

**DOI:** 10.1186/s12896-026-01107-3

**Published:** 2026-02-26

**Authors:** Amira L. Hanna, Atef S. Elgebaly, Magdy M. Mohmed, Mahmoud W. Sadik, Hayam M. Hamouda, Hanan A. Goda, Afaf Altrawy

**Affiliations:** 1 Microbiology Department, Division of Basic Medical Science, Egyptian Drug Authority EDA (National Organization for Drug Control and Research NODCAR), Giza, 12553 Egypt; 2 College of Medical Laboratory Techniques, Al-Farahidi University, Baghdad, 10021 Iraq; 3 Department of Molecular Diagnostics and Therapeutics, Faculty of Biotechnology (Ex. GEBRI), University of Sadat City, Sadat City, 32897 Egypt; 4 Department of Microbiology, Faculty of Agriculture, Cairo University, Giza, 12613 Egypt; 5 Department of Environmental Biotechnology, College of Biotechnology, Misr University for Science and Technology, Giza, Egypt; 6 Department of Medical Biotechnology, Biotechnology College, Misr University for Science and Technology, Giza, Egypt

**Keywords:** Green-synthesized silver nanoparticles, *Cyanobacteria (Desertifilum)*, Ehrlich carcinoma, Apoptosis, *BAX/BCL-2* pathway, Caspase-3, Nanomedicine, In vivo antitumor activity

## Abstract

**Background:**

Green and biogenic silver nanoparticles (AgNPs) are widely researched as anticancer treatments; nevertheless, in vivo data on cyanobacteria-derived formulations and their apoptotic mechanisms are limited.

**Objectives:**

To assess the antitumor activity, hematological safety, and apoptosis-related pathways of *Desertifilum tharense*-derived AgNPs, provided either independently or in conjunction with *Desertifilum* exopolysaccharides (EPS), in a mouse Ehrlich cell carcinoma (ECC) model.

**Methods:**

Male Swiss albino mice were assigned to five groups: healthy control, AgNP toxicity control, tumor control, AgNP-treated tumor, and AgNPs + EPS-treated tumor. Endpoints encompassed body weight, thigh tumor volume, hematological profiling, histology, immunohistochemistry identification of cleaved *caspase 3*, and real-time PCR quantification of *BAX*,* BCL-2*, and *caspase 3* mRNA, utilizing the *BAX/BCL-2* ratio as an indicator of apoptotic equilibrium.

**Results:**

Tumor control subjects exhibited significant increases in body weight and tumor volume, along with anemia, leukocytosis, and altered leukocyte differentials. The AgNP-treated groups had markedly decreased tumor sizes and partial restoration of hematological parameters, whereas healthy mice treated solely with AgNP maintained values within physiological limits. Tumor sections from treated mice exhibited significant necrosis and a treatment-dependent increase in cleaved *caspase 3* staining, most apparent and widespread in the AgNPs + EPS group. Gene expression analysis demonstrated a considerable upregulation of *BAX* and *caspase 3*, alongside a downregulation of *BCL-2* in treated tumors, with the combination formulation exhibiting the highest *BAX/BCL-2* ratio, indicative of pronounced activation of mitochondria-mediated apoptosis.

**Conclusions:**

*Desertifilum*-derived AgNPs demonstrate in vivo antitumor efficacy against ECC with satisfactory hematological safety and induce *caspase 3* dependent, mitochondria-mediated apoptosis via regulation of the *BAX/BCL-2* axis. The co-administration with EPS enhances the pro-apoptotic gene and protein profile, endorsing cyanobacteria-based AgNP/EPS systems as viable candidates for future nano-based cancer therapies.

## Background

Cyanobacteria are a remarkably diversified and highly specialized assemblage of microorganisms adapted to numerous ecological niches. They exist in several ecosystems, including terrestrial, glacial, marine, brackish, and freshwater settings. Furthermore, they can exist independently or in symbiosis with other creatures, and they exhibit considerable resilience to harsh environmental circumstances [1].

Cyanobacteria are an important source of structurally bioactive metabolites, with cytotoxic against various cancer cell lines, including those from colon, lung, and neuroblastoma cancers. These compounds can also show selective toxicity, with some promoting growth in healthy cell lines while inhibiting cancer cell growth [2], antiviral, anticancer, antimitotic, antimicrobial, specific enzyme inhibition, and immunosuppressive activities [3].

The antimicrobial activities and antitumor assays of intracellular and extracellular cyanobacterial extracts were screened. Intracellular extracts exhibited a growth inhibition of 35% for Gram-positive bacteria and 49% for Gram-negative bacteria [4]. *Cylindrospermopsis raciborskii* and *Nostoc sp.* methanolic intracellular extracts also inhibited the growth of cancer cells. *Fischerella sp.* and *Pseudomonas aeruginosa* extracellular extracts inhibited the growth of lung cancer cells. Some species of *Cyanobium* and *Pseudomonas aeruginosa* showed inhibitory effects on colon cancer cells. Because of this, some cyanobacterial strains represent a treasure trove of natural compounds that could find use in biotechnology and pharmaceuticals [3].

These metabolites are potentially rich sources of a vast array of products with biotechnological, agricultural, medical, pharmaceutical and environmental applications [5]. These compounds have diverse biological activities including hypocholesterolemic, anticancer, anti-inflammatory cardio-protective, hepatoprotective, antimalarial, antimicrobial, enzyme inhibiting, immunostimulant, cytotoxic and antioxidant activities that offer rich pharmacological potential [6].

Silver nanoparticles (AgNPs) have been a subject of considerable interest among researchers owing to their tunable properties. The improved characteristics of green synthesized AgNPs can be utilized across various scientific fields and daily applications, including epidemic prevention and the treatment of contagious diseases [7]. The advantages of the biosynthesis method for nanoparticles fabrication over physical and chemical synthesis methods are its less time-consuming, simple, cheap, and eco-friendly nature [8].

Despite growing evidence supporting the in vitro cytotoxic effects of cyanobacteria-derived AgNPs against various cancer cell lines, in vivo investigations remain limited, particularly with respect to their antitumor efficacy, hematological safety, and underlying apoptotic mechanisms in solid tumor models. Notably, whether cyanobacteria-mediated AgNPs activate canonical mitochondrial apoptotic pathways specifically *BAX/BCL-2* signaling in vivo has not been systematically explored.

Therefore, the present study was designed to address this critical knowledge gap by evaluating the in vivo antitumor activity and safety profile of *Desertifilum tharense*-derived AgNPs, administered alone or in combination with cyanobacterial EPS, in a solid Ehrlich carcinoma mouse model. Particular emphasis was placed on elucidating the *BAX /BCL-2* mediated mitochondrial apoptotic pathway, along with downstream *caspase-3* activation, to provide mechanistic insight into the pro-apoptotic effects of cyanobacteria-derived AgNPs. This work aims to bridge the gap between in vitro promise and in vivo mechanistic validation, thereby supporting the potential of green-synthesized AgNPs as a novel anticancer strategy.

## Materials and methods

### Preparation and characterization of cyanobacteria-derived silver nanoparticles

This study expands upon our recent research demonstrating that the cyanobacterial isolates *Phormidium ambiguum* and the newly identified *Desertifilum tharense* effectively facilitate the green manufacture of AgNPs through the biological reduction of Ag⁺ in illuminated environments [9]. This study focused on AgNPs produced extracellularly under light and performed thorough physicochemical characterization before biological evaluation.

Silver nanoparticles used in the present study were synthesized following the identical protocol and experimental conditions previously established by Hanna et al. (2022). The same extracellular reduction method and preparation workflow were employed to ensure reproducibility and consistency of physicochemical properties across batches. This study first conducted a standard physicochemical evaluation of the silver nanoparticles produced by cyanobacteria before proceeding with biological testing. UV–Vis spectroscopy indicated the formation of Ag nanoparticles and surface plasmon resonance, while SEM/TEM illustrated the particles’ size and morphology. XRD confirmed the crystalline structure of silver, and FTIR identified the functional groups of cyanobacteria that capped and stabilized the nanoparticle surface.

### In vivo evaluation of the anticancer activity of cyanobacteria-derived silver nanoparticles against Ehrlich carcinoma in mice

#### Experimental animals and housing

The Experimental Animal House of the national organization for drug control and research (NODCAR), Egyptian drug authority, provided thirty-three male Swiss albino mice, aged 5 to 6 weeks and weighing between 25 and 30 g. Animals were housed in polycarbonate cages with sawdust bedding in a regulated environment (22 ± 2 °C, 12 h of light and 12 h of darkness) and had unrestricted access to a standard pellet diet and tap water. Mice were acclimatized to their new habitat for two weeks prior to the commencement of the trial. All approaches adhered to the ethical guidelines established by the university [10, 11].

#### Ehrlich ascites carcinoma inoculation and experimental design

Tumor cells were collected from a donor mouse bearing a 10-day-old EAC tumor, counted, and diluted with sterile physiological saline at a ratio of 1:10 to prepare a suspension containing 1 × 10⁶ viable cells/mL. Thirty male mice were randomly divided into five groups (*n* = 6). A negative control group consisted of healthy mice without tumor induction. The tumor control group was injected subcutaneously (S.C.) in the right thigh with 0.2 mL of the EAC cell suspension (1 × 10⁶ cells/mL) and left untreated.

Tumor-bearing mice in the treatment groups received intraperitoneal (I.P.) administration of AgNPs at a concentration of 0.05 mM (0.2 mL every 3 days for 3 weeks). The combination treatment group received AgNPs (0.05 mM) in combination with cyanobacterial EPS; 2 g/mL, administered intraperitoneally (I.P.) at a volume of 0.2 mL every 3 days for 3 weeks. An AgNPs toxicity group consisting of healthy mice was administered AgNPs (0.05 mM) intraperitoneally (I.P.) at 0.2 mL every 3 days for 3 weeks to assess potential systemic toxicity in non–tumor-bearing animals.

Body weight was recorded at the beginning and end of the treatment period to provide a basic assessment of health status and tumor burden. At the conclusion of the experiment, all animals were humanely euthanized via cervical dislocation, and tumors along with relevant organs were collected for subsequent biochemical, histological, and molecular analyses.

#### Tumor volume measurement

At the conclusion of the experiment, mice were euthanized, and solid tumors were excised and measured using a Vernier caliper. The tumor volume (V, mm³) was determined from the length (L), breadth (W), and height (H) utilizing the method established by Abd El Ghany et al. (2015).

#### Hematological assessments

Whole blood was collected into EDTA-coated tubes for hematological evaluation. The panel included total white blood cell and red blood cell counts, hemoglobin concentration, and differential leukocyte counts (neutrophils, lymphocytes, monocytes, eosinophils), and blood smears were prepared immediately after sampling for morphological assessment.

#### Histopathology and immunohistochemistry

Tumor specimens underwent fixation in 10% neutral buffered formalin for 48 h, followed by routine dehydration, paraffin embedding, and sectioning at 4 μm. Sections were mounted on glass slides, deparaffinized, and stained with hematoxylin and eosin (H&E) for light microscopic evaluation, following the methodology outlined by El Bialy et al. (2017).

Cleaved *caspase 3* was detected using immunohistochemistry on 5 μm paraffin sections affixed to positively charged slides. Following deparaffinization and rehydration, antigen retrieval was performed in citrate buffer (10 mM, pH 6.0) utilizing microwave heating, subsequently followed by the blocking of endogenous peroxidase and nonspecific binding. Sections were incubated with rabbit monoclonal anti-cleaved *caspase 3* antibody, followed by a biotinylated secondary antibody and streptavidin–HRP (LSAB2 System HRP). Immunoreactivity was visualized using DAB (Dako), and nuclei were counterstained with Mayer’s hematoxylin.

*Caspase 3* immunostaining was evaluated semi-quantitatively by examining a minimum of five high-power fields per tumor and assigning a score from 0 to 3 based on staining intensity and the proportion of positive cells. Score 0 (–) indicates no staining or only faint staining in fewer than 10% of tumor cells. Score 1 (+) signifies weak to moderate staining in 10–20% of cells. Score 2 (++) denotes moderate to strong staining in 21–50% of cells, while score 3 (+++) indicates strong staining in more than 50% of cells. Tumors with scores of 0–1 were classified as having low *caspase 3* expression, while scores of 2–3 indicated high *caspase 3* expression, reflecting an increased level of apoptosis-related signaling.

Malignancy grading was performed using a semi-quantitative scoring system adapted for undifferentiated solid tumors. The evaluation was conducted by two independent observers blinded to the experimental groups. The grading criteria included:​.

1. Nuclear Pleomorphism: Scored as mild (1+), moderate (2+), or marked (3+) based on nuclear size variation, hyperchromasia, and chromatin irregularity.
2. Mitotic Activity: Mitotic figures were counted in 10 consecutive non-overlapping high-power fields (HPF, 400×) within the most active tumor regions.
3. Tumor Necrosis and Response: Therapeutic efficacy was graded based on the extent of coagulative necrosis and viable tumor burden.

#### Quantitative real-time PCR analysis of apoptosis-related genes

Tumor specimens from each experimental group were removed immediately post-euthanasia, rapidly frozen in liquid nitrogen, and preserved at − 80 °C until RNA extraction. Real-time PCR was employed to assess variations in the relative expression levels of *BAX*, *BCL-2*, and *caspase 3 (CASP3)* in tumor specimens across all groups. We used kits from Thermo Scientific in the USA to first isolate total RNA and then turn it into cDNA. Gene-specific primers for *BAX*,* BCL-2*, *caspase3*, and *β-actin* (internal control) were used for qRT-PCR analysis. Primer sequences are provided in Supplementary Table S1. The thermal and melting curve conditions were carried out as previously described [12, 13]. Reactions were performed in duplicates, and relative expression was quantified using the 2^−ΔΔCt^ technique, normalizing target genes to the housekeeping gene and presenting data as fold change relative to the tumor control group. The *BAX /BCL-2* mRNA ratio was derived from the normalized values and used as a measure of the balance between pro-apoptotic and anti-apoptotic signals, with higher ratios indicating more apoptosis in treated tumors.

#### *Insilco*

STRING (https://string-db.org/) was utilized to identify protein-protein interactions and pathways, while Cytoscape (WEB 1.0) was employed to visualize the results. A curated list of apoptosis-related proteins (*Bax*,* Bcl-2*,* CASP3*) was submitted to STRING for Mus musculus. The interactions were founded on experimental and database evidence, exhibiting a high confidence level (combined score > 0.7). We imported the interaction file from STRING into Cytoscape. We modified the edge orientations according to KEGG/Reactome annotations to illustrate activation or inhibition links and to connect upstream death receptor signals to mitochondrial and executioner *caspase* modules.

#### Statistical analysis

Data are presented as mean ± standard deviation (SD) for each group (*n* = 6 mice per group). Differences among experimental groups were assessed using one-way analysis of variance (ANOVA) followed by Dunnett’s post-hoc test (vs. G1 control). For qRT-PCR experiments, relative gene expression was calculated using the 2^^−ΔΔCt^ method, normalized to β-actin, and expressed relative to the tumor control group. Differences were considered statistically significant at *p* < 0.05.

## Results

### Silver nanoparticles characterization

Silver nanoparticles, previously biosynthesized from the extracellular filtrates of *Phormidium ambiguum* and *Desertifilum tharens*e, were verified to be effectively produced and structurally stable. UV–Vis spectroscopy revealed a distinct surface plasmon resonance band between 410 and 450 nm, aligning with the well-dispersed AgNPs generated under illuminated circumstances. Transmission and scanning electron microscopy demonstrated primarily spherical particles organized in loosely aggregated clusters, with particle sizes approximately ranging from 6.24 to 11.7 nm for *D. tharense*-derived AgNPs and from 6.46 to 12.2 nm for *P. ambiguum*-derived AgNPs, indicating nanoscale dimensions appropriate for biological applications. X-ray diffraction patterns displayed the characteristic reflections of a face-centered cubic crystalline silver phase, whereas energy dispersive X-ray investigation verified silver as the predominant elemental constituent. The Fourier transform infrared spectra revealed significant hydroxyl and amide bands, corroborating the function of cyanobacterial biomolecules as natural capping and stabilizing agents on the nanoparticle surface. These physicochemical characteristics are consistent with those previously reported by our group [9], confirming reproducibility of the synthesis procedure and stability of the Desertifilum-derived AgNP formulation used in the current biological experiments.

### In vivo antitumor activity of biosynthesized AgNPs

The extracellularly biosynthesized silver nanoparticles from *Desertifilum tharense* under light conditions were assessed for their in vivo anticancer efficacy in a mouse ECC model. Thirty male Swiss albino mice were randomly allocated into five groups (*n* = 6/group): healthy control mice (G1); untreated tumor-bearing mice (G2); tumor-bearing mice administered AgNPs (0.05 mM; G3); tumor-bearing mice receiving a combination of AgNPs (0.05 mM) and *Desertifilum*-derived EPS; 2 g/mL; G4); and healthy mice treated with AgNPs alone (0.05 mM; G5). The anticancer efficacy was evaluated by tracking body weight and tumor volume, alongside hematological profiling, histological investigation of tumor tissues, and immunohistochemistry assessment of *caspase 3*, which included semi-quantitative scoring of *caspase 3* immunostaining.

**Fig. 1:**
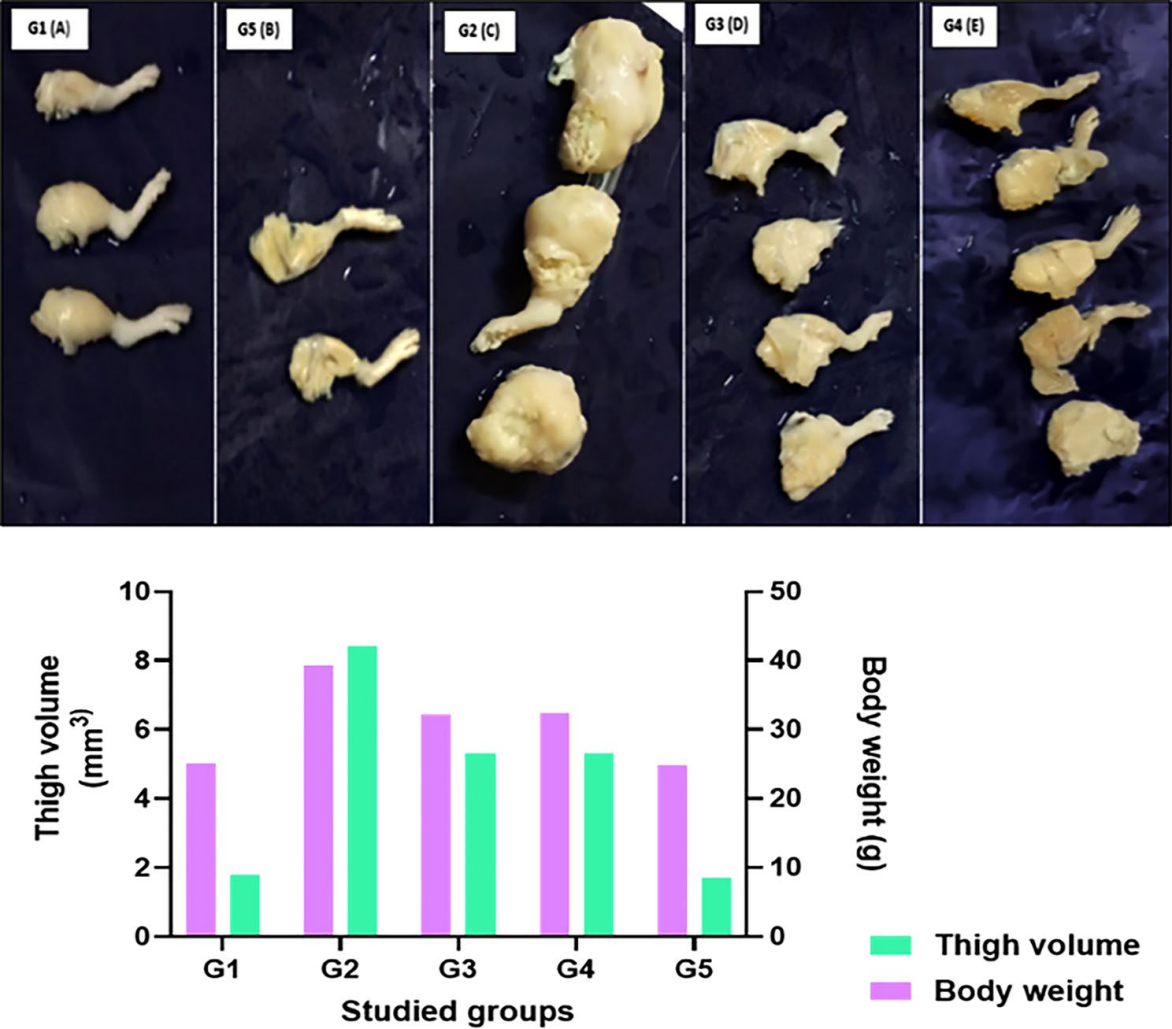
In vivo antitumor efficacy of cyanobacteria-derived AgNPs in the Swiss albino mouse Ehrlich carcinoma model. Top panel (**A**–**E**): Representative macroscopic images of excised thigh tissues/tumors from (**A**) G1 healthy control, (**B**) G5 AgNP toxicity control, (**C**) G2 tumor control, (**D**) G3 AgNP-treated, and (**E**) G4 AgNPs + EPS-treated groups. Bottom panels: quantitative assessment of (**F**) body weight and (**G**) thigh tumor volume at the study endpoint (*n* = 6/group). Data are presented as mean ± SD.The marked reduction in thigh tumor volume in G3 and G4 relative to the tumor control group (G2) indicates a potential antitumor effect of the tested formulations

At the experimental endpoint, untreated mice with tumors (G2) exhibited a significant increase in body weight and thigh tumor volume compared to healthy controls, indicating a considerable tumor burden. Mice administered AgNPs (G3) or AgNPs combined with EPS (G4) demonstrated diminished body weights and decreased thigh volumes compared to the tumor control group, signifying a reduction in tumor growth. As shown in Fig. 1, the mean body weight of healthy control mice (G1) was around 25.1 ± 0.1 g, while AgNPs only mice (G5) had a mean body weight of 24.8 ± 0.4 g, with matching thigh volumes of 1.8 ± 0.5 mm³ and 1.7 ± 0.5 mm³, respectively, indicating the lack of palpable tumors in both groups. In contrast, G2 animals attained a body weight of 39.3 ± 0.5 g with an average thigh volume of 8.4 ± 0.7 mm³, while AgNP treated groups G3 and G4 exhibited intermediate values (32.2 ± 2.4 g and 32.3 ± 3.4 g; 5.3 ± 2.2 mm³ and 5.3 ± 2.6 mm³, respectively), indicative of a partial yet significant inhibition of ECC progression by the evaluated formulations.

The highest body weight (39.3 ± 0.5 g) and thigh tumor volume (8.4 ± 0.7 mm³) were observed in the tumor control group (G2), indicating substantial tumor burden. In contrast, tumor-bearing mice treated with Ag-NPs alone (G3) or with Ag-NPs plus EPS (G4) showed markedly lower body weights (32.2 ± 2.4 and 32.3 ± 3.4 g, respectively) and reduced thigh volumes (5.3 ± 2.2 and 5.3 ± 2.6 mm³, respectively), with no appreciable difference between the two treated groups.

### Hematological parameters

Hematological investigation indicated notable changes in the blood profile of tumor-bearing mice relative to healthy controls (Table 1). The tumor control group (G2) had a significant reduction in hemoglobin concentration (11.3 g/dL) and red blood cell count (4.21 × 10⁶/µL) compared to the healthy control group (G1; 17 g/dL and 5.42 × 10⁶/µL, respectively), indicating of tumor-associated anemia. In contrast, total white blood cell (WBC) counts were markedly increased in G2 (34,250 cells/µL) compared to G1 (8,250 cells/µL), indicating a systemic leukocytosis often linked to tumor growth and inflammation. Treatment with AgNPs, whether administered alone (G3) or in conjunction with EPS (G4), significantly improved these hematological abnormalities. In the AgNP treated group (G3), WBC counts approached normal levels (8,500 cells/µL), equivalent to the negative control (G1).

Likewise, the AgNPs + EPS group (G4) exhibited a significant decrease in leukocytosis (10,500 cells/µL) relative to the untreated tumor group. The toxicity control group (G5), administered AgNPs only, preserved hematological parameters within the normal physiological range (WBC: 6,000 cells/µL; Hb: 18 g/dL), so affirming the biocompatibility and absence of systemic hematotoxicity of the biosynthesized nanoparticles at the evaluated dosage. The differentiation of the leukocyte population (Table 1) further emphasized the immunomodulatory effects of the therapies. Tumor induction (G2) correlated with an increase in neutrophilia (47%) and a relative decrease in lymphocytes compared to healthy individuals. Treatment with AgNPs (G3) and AgNPs + EPS (G4) altered these ratios, with G3 exhibiting a neutrophil/lymphocyte profile (58% neutrophils, 31% lymphocytes) that coincided with the remission of total leukocytosis.

Crucially, the AgNP toxicity control group (G5) showed no statistically significant deviations in RBC counts, hemoglobin levels, or total WBC counts compared to the healthy control group (G1). Coupled with the observation of normal body weight gain in this group (Table 1), these findings suggest that the administered dose of AgNPs does not induce overt systemic toxicity or myelosuppression in non-tumor-bearing hosts.

**Table 1:** Hematological profile of five experimental groups (*n* = 6 per group) at the study endpoint

Group	Hb (g/dL)	RBCs (10¹²/L)	WBCs (cells/µL)	Differential Leukocyte Count (%)			
				Neutrophils	Lymphocytes	Monocytes	Eosinophils
G1 (Healthy control)	17.0 ± 0.8	5.42 ± 0.4	8,250 ± 540	21 ± 3	32 ± 4	4 ± 1	3 ± 1
G2 (Tumor control)	11.3 ± 1.1*	4.21 ± 0.5*	34,250 ± 2,100*	47 ± 5*	45 ± 4*	6 ± 1	2 ± 1
G3 (AgNPs treated)	16.0 ± 1.2#	5.39 ± 0.6#	8,500 ± 620#	58 ± 4#	31 ± 3#	6 ± 1	5 ± 1
G4 (AgNPs + EPS)	13.0 ± 0.9#	4.14 ± 0.4	10,500 ± 850#	55 ± 6#	36 ± 5#	5 ± 1	4 ± 1
G5 (AgNPs toxicity)	18.0 ± 0.7	5.68 ± 0.3	6,000 ± 480	66 ± 5	28 ± 4	3 ± 1	3 ± 1

Data are presented as Mean ± SD for six mice per group (*n* = 6). Statistical analysis was performed using one-way ANOVA; * indicates a significant difference vs. the healthy control group (G1) at *p* < 0.05.

# indicates a significant difference from the untreated tumor control group (G2) at *p* < 0.05.

### Histopathological evaluation of antitumor efficacy

**Fig. 2:**
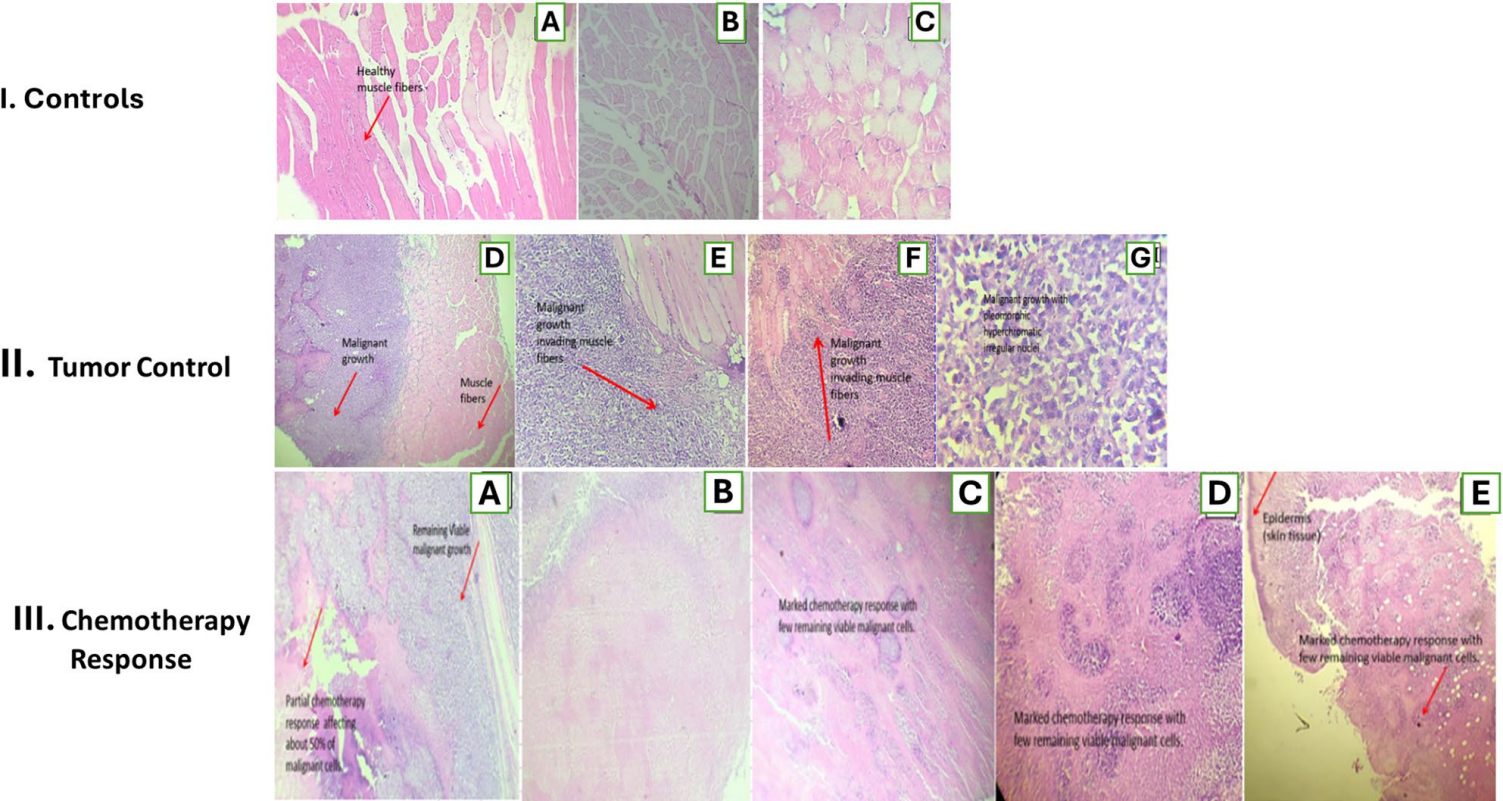
Histopathological features of control, tumor, and treated groups (H&E staining, *n* = 6 mice/group). Row I (Controls): (**A**) healthy skeletal muscle from G1 with normal fibers and peripheral nuclei (40×); (**B**, **C**) G5 (Ag-NPs only) showing mild fiber atrophy and focal myositis without tumor infiltration (**B**: 40×; C: 200×). Row II (Tumor control): (**D**–**F**) G2 displaying extensive malignant growth replacing and invading muscle fibers (40×); (**G**) high-power view of pleomorphic, hyperchromatic tumor cells (200×). Row III (Chemotherapy response): (**A**) G3 (Ag-NPs) with partial response and residual viable tumor (40×); (**B**–**E**) G4 (Ag-NPs + EPS) showing marked chemotherapy response with widespread tumor necrosis and few remaining malignant cells in muscle and overlying skin (40×)

Histopathological investigation of thigh muscle sections revealed diverse morphological patterns throughout the experimental groups (Fig. 2). In the healthy control group (I-A), skeletal muscle fibers exhibited normal architecture, characterized by uniform diameter, preserved striations, and nuclei located peripherally, so confirming tissue integrity.

The toxicity control group (I-B, C), administered solely Ag-NPs, exhibited mild myopathic changes, including atrophied muscle fibers with rounded edges and localized myositis. This indicates that the tissue exhibited minimal responsiveness to the nanoparticle composition. In contrast, the untreated tumor control group (II-D–G) displayed features of high-grade malignancy, characterized by marked nuclear pleomorphism (Score 3+), a high mitotic index (> 5 figures/HPF), and aggressive invasion into surrounding muscle bundles.

The malignant cells exhibited significant pleomorphism and hyperchromatic, irregular nuclei (II-G), and it was evident that they were infiltrating adjacent muscle bundles (II-E, F). The delivery of Ag-NPs (III-A) produced a moderate chemotherapeutic response, evidenced by localized necrotic areas constituting approximately 50% of the tumor mass, despite the presence of viable malignant clusters. Nonetheless, the simultaneous injection of Ag-NPs and EPS (III-B–D) elicited a marked anticancer response, characterized by extensive coagulative necrosis and a substantial reduction in viable tumor burden inside the muscle tissue. The examination of the overlying skin in this group (III-E) demonstrated significant tumor shrinkage with minimal residual malignant cells, underscoring the superior therapeutic efficacy of the combination formulation relative to Ag-NPs alone.

### Activation of *caspase 3* mediated apoptosis by AgNPs

**Fig. 3:**
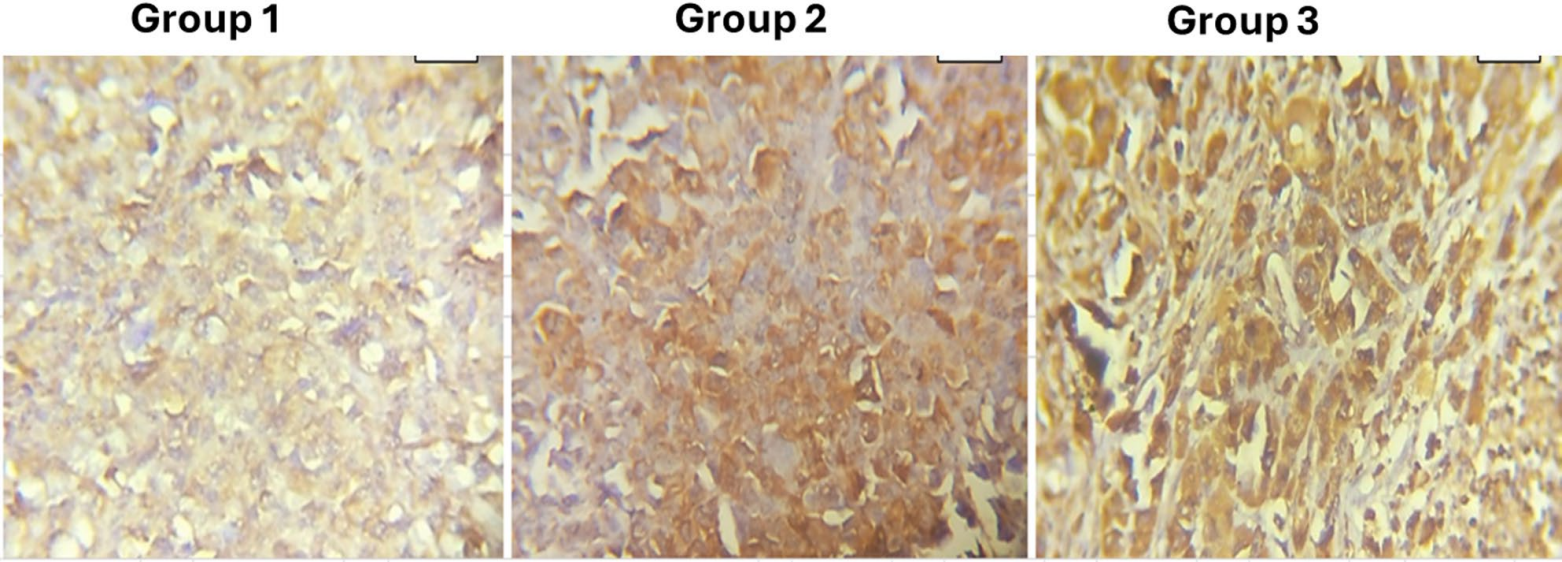
Immunohistochemical expression of cleaved *caspase 3* in Ehrlich carcinoma. Representative tumor sections from **group 1** (untreated control), **group 2** (Ag-NPs), and **group 3** (Ag-NPs + EPS) showing a treatment-dependent increase in cytoplasmic *caspase 3* staining intensity and extent, with weak expression in group 1, stronger patchy positivity in group 2, and strong diffuse positivity in group 3. All images: 400x magnification

Immunohistochemical labeling for cleaved *caspase 3* demonstrated a distinct, treatment-dependent elevation in apoptotic activity within Ehrlich tumor cells (Fig. 3, groups 1–3). In group 1 (untreated tumor control), malignant cells displayed minimal, coarsely granular cytoplasmic *caspase 3* staining, signifying low basal activation. In group 2 (AgNPs alone), cytoplasmic *caspase 3* staining was significantly more pronounced and included a greater proportion of tumor cells, indicating a moderate induction of apoptosis. Group 3 (AgNPs + EPS) exhibited the most significant effect, characterized by intense, diffuse cytoplasmic positivity for *caspase 3* in the majority of malignant cells, indicating strong and uniform activation of the *caspase 3* dependent apoptotic pathway and affirming the enhanced pro-apoptotic efficacy of the combined treatment. Immunohistochemistry for cleaved caspase-3 showed weak cytoplasmic expression in untreated tumors (G2; Score 1, +), stronger but heterogeneous/patchy cytoplasmic expression in Ag-NP–treated tumors (G3; Score 2, ++), and strong diffuse cytoplasmic expression in Ag-NP + EPS–treated tumors (G4; Score 2, ++), indicating increased activation of the executioner caspase pathway following treatment.

### Apoptosis gene signature in response to AgNP based treatments

The relative mRNA expression levels of *BAX*, *BCL-2*, *caspase-3*, and the *BAX/BCL-2* ratio were quantified by qRT-PCR in tumor tissues from the experimental groups: G1 (healthy control), G2 (tumor control), G3 (AgNPs-treated), and G4 (AgNPs + EPS-treated). Data are presented as mean fold change relative to the healthy control group.

**Fig. 4:**
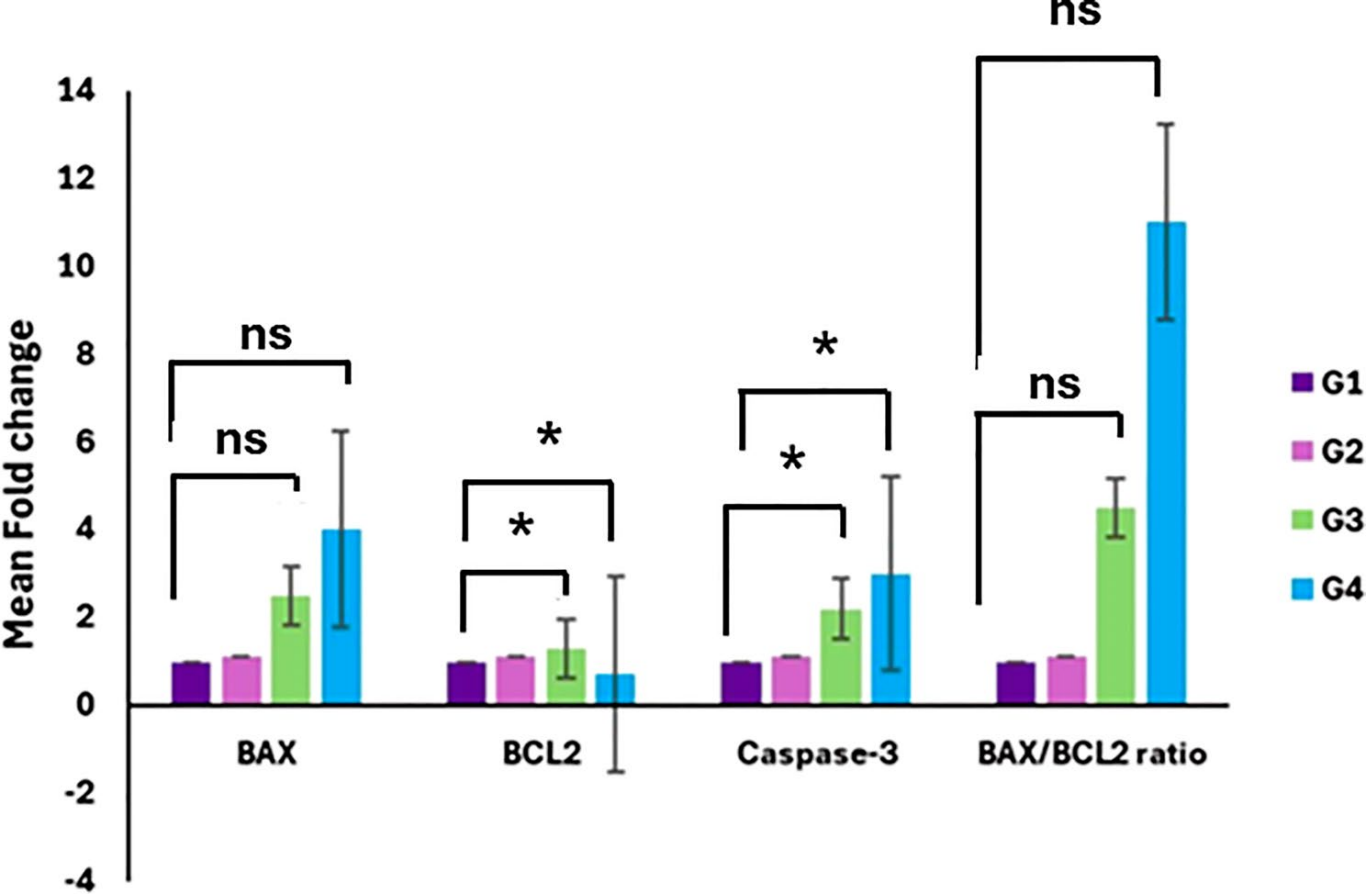
Relative mRNA expression levels of *Bax*,* BCL-2*, *caspase-3*, and the *Bax/BCL-2* ratio were quantified by qRT-PCR in tumor tissues from G1 (healthy control), G2 (tumor control), G3 (AgNPs-treated), and G4 (AgNPs + EPS-treated) groups. Data are expressed as mean fold change relative to the healthy control group and presented as mean ± SD (*n* = 4). Statistical analysis was performed using a one-way ANOVA followed by Dunnett’s post-hoc test. Asterisks (*) denote *p* < 0.05 vs. G1; ns, not significant vs. G1

As illustrated in Fig. 4, *BAX* expression remained low in the healthy and tumor control groups, while treatment with AgNPs (G3) induced an increase that was further enhanced in the AgNPs + EPS group (G4). Although this pattern indicates a treatment-associated upregulation of *BAX*, the change did not reach statistical significance (*p* = 0.0704).

In contrast, *BCL-2* expression was elevated in the tumor control group compared with healthy controls but was significantly reduced following treatment with AgNPs alone or in combination with EPS, reflecting suppression of anti-apoptotic signaling (*p* = 0.0126).

Consistently, *caspase-3* expression was markedly upregulated in the treated groups, with the highest levels observed in G4. This increase reached statistical significance (*p* = 0.0466), indicating activation of the executioner phase of apoptosis.

The *BAX/BCL-2* ratio, a key indicator of mitochondrial apoptotic balance, exhibited a pronounced increase in response to treatment, particularly in the AgNPs + EPS group. However, this change did not reach statistical significance (*p* = 0.1516).

Collectively, these findings demonstrate that AgNPs treatment, especially in combination with EPS elicits a pro-apoptotic transcriptional response in Ehrlich carcinoma tumors, characterized by significant downregulation of *BCL-2* and activation of *caspase-3*, accompanied by a consistent trend toward increased *BAX* expression and *BAX/BCL-2* ratio.

**Fig. 5:**
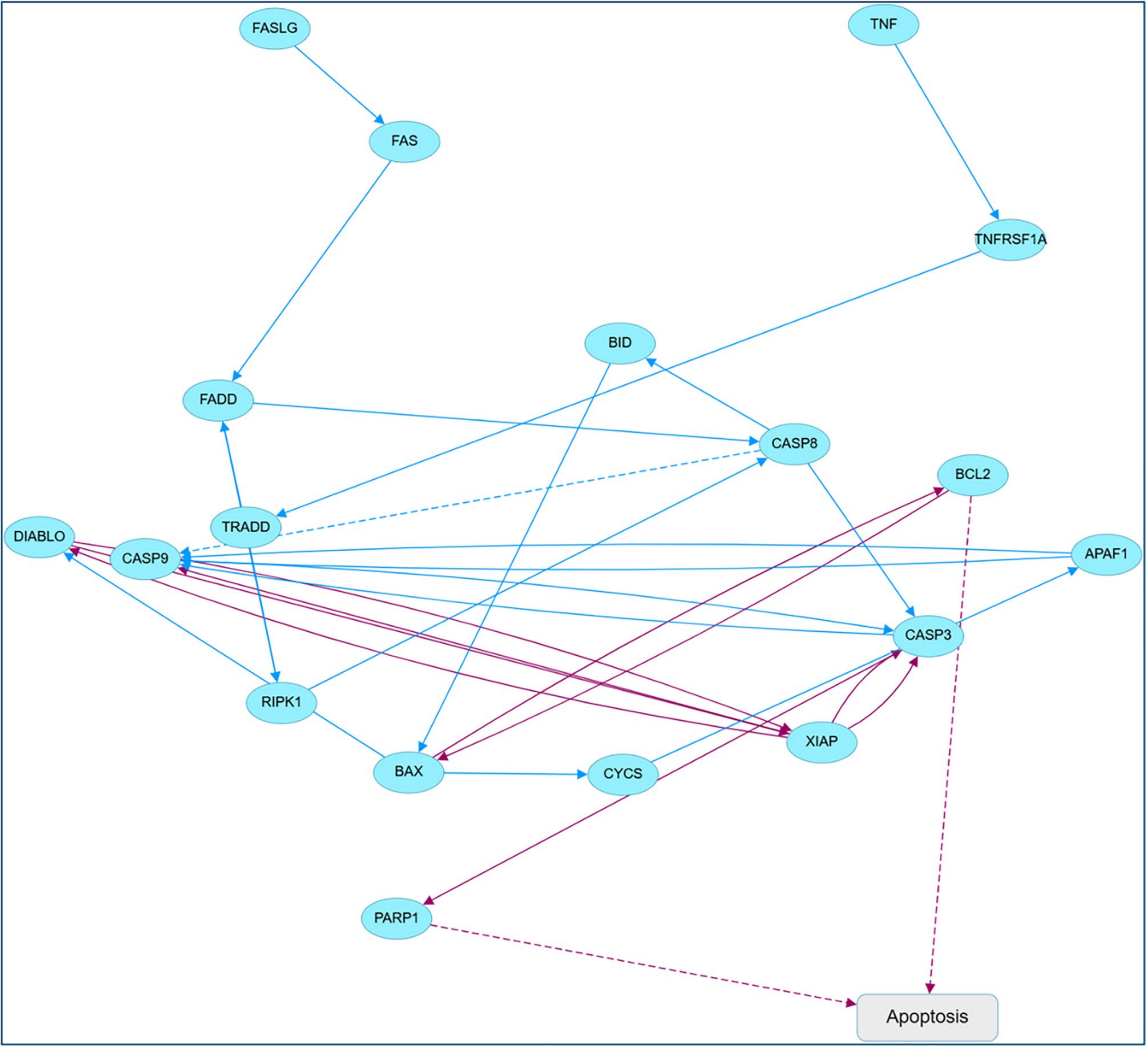
Predicted apoptotic signaling network affected by AgNPs and AgNPs + EPS treatment. Pathway analysis was performed using apoptosis-related differentially expressed genes identified in the present study (*BAX*,* BCL2* and *CASP3*) and mapped onto the canonical Fas/FasL- and TNF-mediated apoptotic cascades together with mitochondrial (intrinsic) pathway components. Blue nodes represent upstream receptors, adaptors and mitochondrial regulators, whereas purple nodes and arrows highlight the experimentally modulated genes and the predicted downstream propagation of their effects toward *caspase-3* activation and execution of apoptosis. Solid arrows indicate activating interactions, and dashed arrows indicate indirect or predicted regulatory relationships. All gene symbols follow official nomenclature, APS, apoptosis

The STRING–Cytoscape network (Fig. 5) illustrates that TNF and FAS ligands engage TNFRSF1A, TRADD, and FADD to activate *CASP8*, which subsequently either directly activates *CASP3* or transmits signals through BID to the mitochondrial route. *Bax* and *Bcl-2* are located in the mitochondrial checkpoint, overseeing *CYCS* release, *APAF1–CASP9* apoptosome assembly, and subsequent C*ASP3* activation, whilst *DIABLO* and *XIAP* influence caspase activity and the *PARP1* cleavage pathway leading to apoptosis. This map positions the experimentally quantified genes (*BAX*,* BCL-2*,* Caspase 3*,* Caspase 9*) as pivotal nodes where extrinsic and intrinsic apoptotic pathways intersect.

## Discussion

This study presents in vivo evidence that silver nanoparticles produced from cyanobacteria demonstrate significant anticancer activity against Ehrlich cell carcinoma while preserving a satisfactory safety profile in healthy mice. This study sought to determine if biosynthesized silver nanoparticles, either independently or in conjunction with *Desertifilum-*derived extracellular polymeric substances, can influence tumor growth and apoptosis in a clinically relevant murine model by integrating traditional macroscopic endpoints (body weight and tumor volume) with hematological, histopathological, immunohistochemical, and molecular analyses. This integrative strategy is crucial as numerous nanomaterials exhibit significant cytotoxicity in vitro yet do not attain substantial tumor control in vivo or are constrained by systemic toxicity [14, 15].

The present findings show that untreated tumor-bearing mice experienced large increases in body weight and thigh tumor volume, indicating fast, space-occupying solid Ehrlich carcinoma growth. In contrast, mice given AgNPs alone or with EPS had significantly lower body weights and thigh volumes than the tumor control group, indicating that both formulations inhibited ECC growth. Healthy control mice and animals treated with AgNPs alone maintained near-normal body weight and had no palpable thigh masses, indicating that the tested nanoparticle dose did not cause systemic toxicity. The macroscopic antitumor effects of AgNPs and the AgNPs + EPS groups were similar in body weight and tumor volume, but histopathology, *caspase 3* immunohistochemistry, and *BAX/BCL-2/caspase 3* gene expression suggest that EPS increases the nanoparticles’ pro-apoptotic effect at the cellular and molecular levels, even if it doesn’t always shrink the tumor. These findings suggest that biogenic AgNPs may treat ECC and that judicious combinations with bioactive polysaccharides may make them more powerful against tumors while being safe.

The induction of tumors via ECC resulted in a typical cancer-associated hematological profile, characterized by anemia, leukocytosis, and alterations in white blood cell composition. The notable reduction in Hb and RBCs, coupled with a marked increase in total WBCs in the tumor control group (G2) relative to healthy mice (G1), is consistent with previous research on Ehrlich carcinoma–bearing mice, indicating that tumor burden results in anemia of chronic disease and inflammatory leukemoid responses. Treatment with biosynthesized AgNPs, both alone (G3) and in combination with EPS (G4), notably corrected these abnormalities: WBC counts approached normative levels, and erythrogram parameters improved, indicating a decrease in tumor-induced systemic stress rather than an increase in myelotoxicity. Comparable normalization of hematological indices after AgNP-based therapy has been documented for biogenic and cyanobacteria-derived AgNPs in EAC models, supporting the hematological safety and anticancer efficacy of these formulations. The maintenance of normal or slightly elevated counts in the AgNP toxicity group (G5) supports the conclusion that the administered dose was well tolerated. This finding is consistent with recent studies suggesting that green synthesized AgNPs can be designed to provide substantial antineoplastic effects while reducing negative impacts on blood parameters in vivo [16, 17].

The *caspase 3* IHC pattern strongly indicates that biosynthesized AgNPs induce apoptosis in ECC cells, with EPS amplifying this effect. The subtle, coarsely granular cleaved *caspase 3* staining in untreated tumors (G 1) is indicative of rapidly proliferating Ehrlich tumors, demonstrating minimal basal apoptotic activity relative to mitotic activity. After AgNP treatment (G 2), the notable increase in cytoplasmic *caspase 3* positive cells, despite a heterogeneous distribution, indicates the initiation of the execution phase of apoptosis in a substantial fraction of the neoplastic population. This aligns with other studies in Ehrlich and other tumor models indicating that green or biogenic AgNPs trigger *caspase 3* activation and apoptotic cell death, often linked to DNA fragmentation and histological necrosis [18, 19].

The gene expression profile reveals that AgNPs produced from *Desertifilum*, especially when combined with EPS, primarily induce their anticancer action via mitochondria-mediated apoptosis. The slight upregulation of *BAX* and *caspase 3*, along with the sustained overexpression of *BCL-2* in the untreated tumor group, illustrates the established imbalance favoring prosurvival signaling in Ehrlich carcinoma and other aggressive malignancies, where intrinsic apoptotic mechanisms are minimally activated. Conversely, AgNP treatment alone substantially altered this equilibrium by considerably elevating *BAX* and *caspase 3* levels while diminishing *BCL-2*, leading to a pronounced increase in the *BAX/BCL-2* ratio, a recognized molecular indicator of commitment to mitochondrial death. Comparable *BAX/BCL-2/caspase-3* signatures have been documented for biogenic and green-synthesized silver nanoparticles in Ehrlich and solid tumor models, wherein the nanoparticles augmented pro-apoptotic gene expression, increased the *BAX/BCL-2* ratio, and simultaneously diminished tumor burden [18, 20, 21].

The integrated AgNPs + EPS formulation exhibited the most significant pro-apoptotic profile, characterized by elevated *BAX* and *caspase 3* mRNA levels, reduced *BCL-2* expression, and, as a result, the highest *BAX/BCL*-2 ratio across all groups. The enhancement of apoptosis-related gene responses aligns with research on polysaccharide-capped or biologically coated metal nanoparticles, wherein natural polymers augment nanoparticle stability, cellular uptake, and oxidative or mitochondrial stress, thereby facilitating intrinsic apoptosis instead of functioning as primary cytotoxins. The strong correlation among the real-time PCR profile, the diffuse cleaved *caspase 3* immunostaining pattern, and the significant histological necrosis observed in treated tumors substantiates a coherent mechanistic model. This model posits that *Desertifilum-*derived AgNPs, particularly when combined with EPS, promote ECC regression by reprogramming the *BAX/BCL-2* axis and activating the *caspase 3* execution pathway, consistent with contemporary mechanistic theories for nano-enabled anticancer therapies utilizing silver and other biogenic nanoparticles [22, 23].

The integration of STRING and Cytoscape network analysis with our real-time PCR and immunohistochemistry data suggests that *Desertifilum*-derived AgNPs modify numerous nodes within the apoptotic interactome instead of targeting a singular entity. The observed upregulation of *BAX* and *caspase 3*, downregulation of *BCL-2*, and increased *BAX/BCL-2* ratio position AgNPs (± EPS) at the mitochondrial decision point of this network, facilitating *CYCS/APAF1–CASP9* activation and alleviating XIAP-mediated inhibition of *CASP3*, consistent with established apoptosis networks for biogenic AgNPs. This STRING–Cytoscape generated map offers a molecular framework that substantiates the hypothesis that the evaluated nanoformulations induce ECC regression by synchronously activating death receptor and mitochondrial pathways that converge on *caspase 3*-dependent apoptosis [16].

In summary, this study demonstrates that silver nanoparticles derived from *Desertifilum*, especially when combined with EPS, exhibit significant antitumor effects against Ehrlich cell carcinoma while ensuring acceptable hematological safety. The combined evidence from decreased tumor burden and the rectification of tumor-induced hematological disturbances, alongside *caspase 3* activation and a *BAX/BCL-2/caspase 3* gene expression profile, suggests that these biosynthesized AgNPs primarily function through mitochondria-mediated apoptosis rather than through nonspecific cytotoxicity. This mechanistic pattern aligns with recent studies on green and biogenic AgNPs, emphasizing the roles of ROS generation, mitochondrial damage, and the modulation of the *BAX/BCL-2* axis and *caspase* cascades as key factors in their anticancer activity. The data underscores the necessity for additional research focused on dose optimization, long-term toxicity, pharmacokinetics, and potential combinatorial strategies prior to the feasible translation of this nano system into clinical application.

Comparative studies with commercial AgNPs and non-cyanobacterial AgNPs will determine whether *Desertifilum*-derived capping agents confer superior tumor targeting, circulation time, or apoptotic pathway specificity. Furthermore, a limitation of this study is the absence of in vitro experiments, which are needed to further dissect the precise molecular mechanisms of *Desertifilum* derived AgNP induced apoptosis.

While the current study provides robust evidence of AgNP-induced apoptosis through *caspase-3* activation, *BAX/BCL-2* modulation, and histological necrosis; direct apoptotic index quantification via TUNEL staining or Annexin V/PI flow cytometry which were not performed here would strengthen mechanistic validation and is planned for future dose-response studies.

Despite the robust apoptosis validation provided by histopathology, cleaved *caspase-3 *immunohistochemistry, and gene-expression profiling, several limitations should be acknowledged. Protein-level assessment in the present study relied primarily on immunohistochemical detection rather than western blot analysis. While immunohistochemistry offers the advantage of preserving tissue architecture and spatial context allowing localization of apoptotic cells within heterogeneous solid tumors whole-tissue protein quantification methods such as western blotting may provide additional complementary confirmation. Therefore, future mechanistic studies, particularly in controlled in vitro or cell line models, will incorporate western blo based protein quantification to further strengthen pathway validation.

## Conclusion

This study demonstrates that biosynthesized silver nanoparticles derived from *Desertifilum tharense* exhibited a significant antineoplastic effect on ECC while maintaining stable blood cell levels at the studied dosage. Treatment with AgNPs, particularly in conjunction with appropriate exopolysaccharides, decreased tumor size, ameliorated anemia and leukocytosis associated with tumors, and induced significant histological necrosis. The concurrent increase in cleaved *caspase 3* immunoreactivity and the levels of *BAX* and *caspase 3* transcripts, together with the downregulation of *BCL*-*2* and an elevated *BAX/BCL-2* ratio, indicates that these formulations promote mitochondria-mediated apoptosis rather than nonspecific cytotoxicity. Current research on green and biogenic silver nanoformulations indicates that *Desertifilum-*derived AgNPs, whether used independently or with EPS, represent a promising basis for future preclinical optimization, including dose-response studies, pharmacokinetic evaluations, and long-term safety assessments, before any potential translational application.

## Data Availability

All data generated or analysed are included in the current MS.
